# Direct protein quantification in complex sample solutions by surface-engineered nanorod probes

**DOI:** 10.1038/s41598-017-04970-5

**Published:** 2017-07-06

**Authors:** Stefan Schrittwieser, Beatriz Pelaz, Wolfgang J. Parak, Sergio Lentijo-Mozo, Katerina Soulantica, Jan Dieckhoff, Frank Ludwig, Joerg Schotter

**Affiliations:** 1 Molecular Diagnostics, AIT Austrian Institute of Technology, Vienna, Austria; 2 Fachbereich Physik, Philipps-Universität Marburg, Marburg, Germany; 3 CIC Biomagune, San Sebastian, Spain; 4 Laboratoire de Physique et Chimie des Nano-objets (LPCNO), Université de Toulouse; INSA, UPS, CNRS, Toulouse, France; 5 Institute of Electrical Measurement and Fundamental Electrical Engineering, TU Braunschweig, Braunschweig, Germany; 6 Centro Singular de Investigación en Química Biológica y Materiales Moleculares (CiQUS) y Departamento de Física de Partículas, Universidade de Santiago de Compostela, Santiago de Compostela, Spain; 7 NABLA Lab, Biological and Environmental Sciences and Engineering (BESE) Division, King Abdullah University for Science and Technology (KAUST), Thuwal, 23955-6900 Saudi Arabia; 8 Diagnostic and Interventional Radiology Department and Clinic, University Medical Center Hamburg-Eppendorf, Hamburg, Germany

## Abstract

Detecting biomarkers from complex sample solutions is the key objective of molecular diagnostics. Being able to do so in a simple approach that does not require laborious sample preparation, sophisticated equipment and trained staff is vital for point-of-care applications. Here, we report on the specific detection of the breast cancer biomarker sHER2 directly from serum and saliva samples by a nanorod-based homogeneous biosensing approach, which is easy to operate as it only requires mixing of the samples with the nanorod probes. By careful nanorod surface engineering and homogeneous assay design, we demonstrate that the formation of a protein corona around the nanoparticles does not limit the applicability of our detection method, but on the contrary enables us to conduct *in*-*situ* reference measurements, thus further strengthening the point-of-care applicability of our method. Making use of sandwich assays on top of the nanorods, we obtain a limit of detection of 110 pM and 470 pM in 10-fold diluted spiked saliva and serum samples, respectively. In conclusion, our results open up numerous applications in direct protein biomarker quantification, specifically in point-of-care settings where resources are limited and ease-of-use is of essence.

## Introduction

Molecular diagnostics employed for the diagnosis and prognostics of a wide range of diseases is based on the detection of biomarkers in complex sample solutions and is of enormous scientific and clinical interest^1, 2^. Within the range of methods employed for biomarker detection, homogenous measurement techniques are of special relevance for point-of-care (PoC) testing settings, as they allow for omitting complex sample preparation steps. Thus, the time for sample analysis may be reduced, whilst ensuring at the same time maximal ease-of-use^3^. Here, magnetic nanoparticles play a major role due to their added capability of magnetic manipulation, which can be exploited, for example, to accelerate binding processes or to enhance the signal to noise ratio^4^. Alternative non-magnetic nanoparticle-based bio sensing techniques include surface-enhanced Raman spectroscopy^5, 6^ or methods relying on fluorescent nanoparticle properties^7^. It has been shown that the combination of nanoparticle labels and surface-enhanced Raman spectroscopy allows to detect various biomarkers in complex sample solutions^8, 9^.

In the present article, we show the applicability of our previously introduced magnetic nanoparticle-based homogeneous measurement principle to molecular diagnostics in complex samples, i.e. serum and saliva samples. The method relies on changes of the hydrodynamic nanoparticle volume upon analyte molecule binding. To that end, antibody-functionalized magnetic nanorods (‘nanoprobes’) are excited in solution by a rotating magnetic field (RMF), which results in a rotational nanoprobe motion. The hydrodynamic volume of the nanoprobes induces a rotational drag torque with the result that the nanoprobes lag behind the RMF by a characteristic phase lag. Binding of the antigen causes an increase of the hydrodynamic nanoprobe volume, which can be observed directly via a change of the phase lag. This effect can be further enhanced by also adding secondary antibodies to form a sandwich-type immunoassay on top of the nanoprobe surface. The phase lag is determined optically by measurements of the actual nanoprobe alignment. This is made possible by the elongated nanoparticle geometry that causes anisotropic absorption and scattering. When applying linearly polarized incident light, this effect allows for deducing the actual nanoprobe orientation in the sample solution via transmission measurements. By correlating the measured actual nanoprobe orientation with the momentary vector of the applied RMF, the phase lag angle can be determined, and our signal is defined by the change in phase lag angle (Δα) between the sample and a suitable reference 10–12. Next to the inherent advantages of homogenous magnetic nanoparticle-based measurement methods, we show that our method is capable of determining quantitative biomarker concentration levels in complex samples by *in*-*situ* referencing.

As model protein we have chosen the soluble domain of the human epidermal growth factor receptor 2 - sHER2, which is the extracellular domain of HER2, a receptor-like tyrosine kinase that is reported to be involved in several types of human carcinomas^13^. The extracellular sHER2 protein is shed into the blood stream so that it can be found in serum as well as in saliva samples, currently being mainly of interest for the diagnosis as well as for the prognosis of breast cancer^14, 15^. Currently, the clinical cut-off value for sHER2 in serum is 170 pM^14^, while the clinical cut-off value for saliva is one order of magnitude below the serum value^15^.

In the following sections, we describe the measures that are taken to detect the sHER2 analyte protein in complex samples of serum and saliva, which were spiked with sHER2. These measures include the right choice of type and concentration of secondary antibodies as well as the dilution factor of the complex sample solutions. Finally, we conclude by summarizing the major results and by giving an outlook on how to further apply and improve the measurement method.

## Results and Discussion

**Figure 1:**
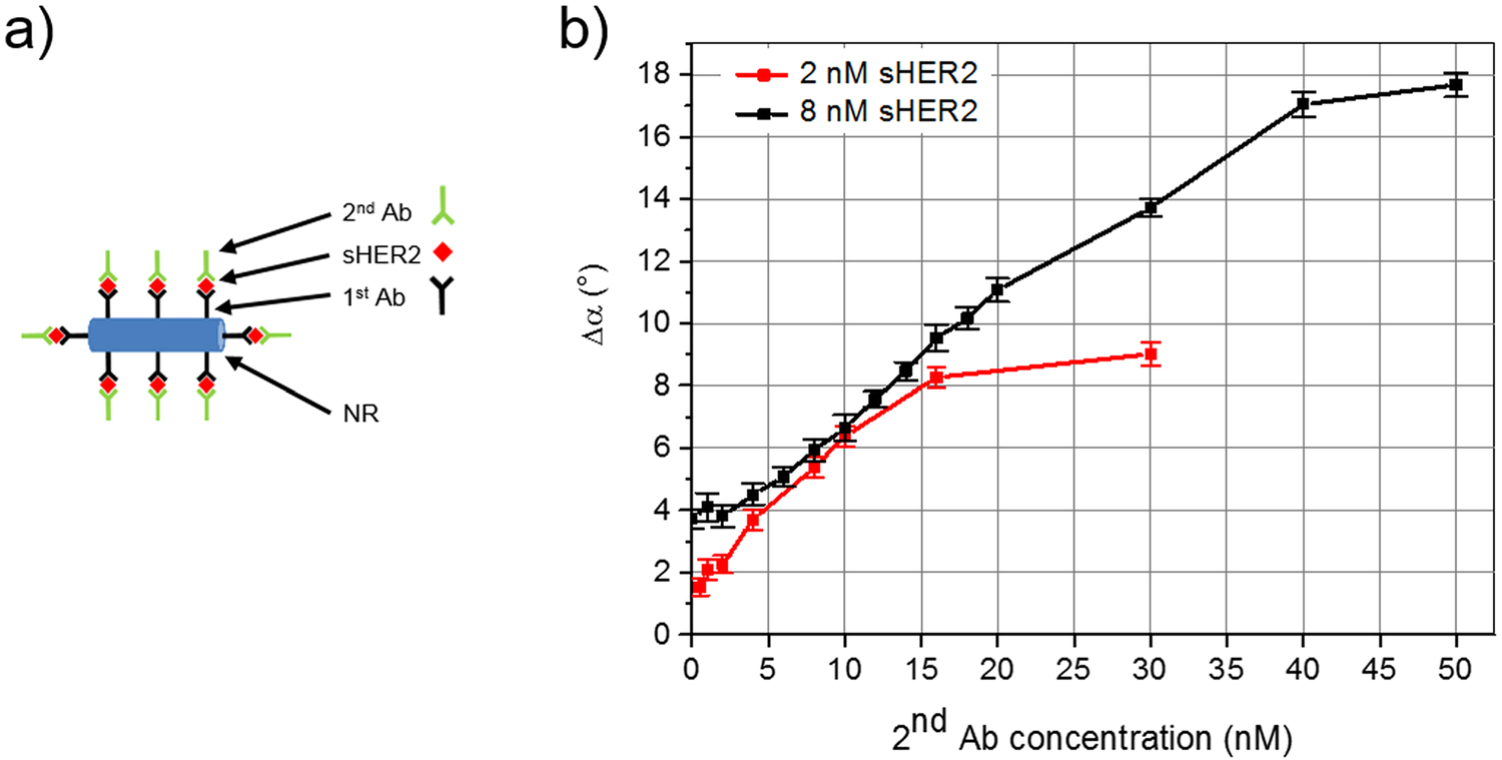
Signal dependence on secondary antibody concentration. (**a**) Sketch of the nanorod (NR) based sandwich-type immunoassay employing primary (1^st^) and secondary (2^nd^) antibodies (Ab); (**b**) Phase lag difference Δα in measurement buffer solution for two fixed concentrations of sHER2 analyte molecules (2 and 8 nM) in dependence of the added concentration of 2^nd^ antibodies.

Simplest mix-and-measure detection of sHER2 analyte in complex solution spiked with sHER2 could be executed by adding the nanoprobes to the sample solution, followed by determining the phase lag difference Δα with respect to a reference sample. To that end, initial measurements have been executed in 10-fold diluted serum samples. What at a first glance seemed to be a promising measurement approach did not result in an analyte molecule concentration-dependent signal (see Supplementary Fig. S1). This we attribute to the formation of a protein corona in complex samples surrounding the nanoprobe surface^16^, which screens the measurement effect of bound analyte molecules. Hence, additional measures had to be taken to gain specificity. This can be achieved by employing secondary antibodies (2^nd^ Abs) in a sandwich-type immunoassay format (see sketch in Fig. 1a). The 2^nd^ Abs attach to the nanoprobe-bound analyte molecules, and, when extending the protein corona thickness, retrieve the concentration dependent measurement signal that has previously been observed for spiking analyte molecules alone into buffer solutions^10^. The exact composition and structure of the protein corona formed on top of the nanoprobes cannot be answered here, while in principle we assume a single layer of adsorbed proteins. Detailed studies applying exactly the same conditions will be required to gain a deep understanding of the fundamental principles of protein corona formation for our nanoprobes.

To characterize the effect of adding 2^nd^ Abs to our measurement technique, we first determined their optimal concentration. Figure 1b shows the dependence of the measurement signal in buffer solution of nanoprobes functionalized by the primary antibody Ab-a on adding varying concentration of the secondary antibody Ab-b for two distinct concentrations of sHER2 analyte molecules (2 nM and 8 nM). Clearly, the signal increased for rising 2^nd^ Ab concentrations up to a saturation level, which correlates with the concentration of analyte molecules. Specifically, the signal saturated for both analyte concentrations when adding at least five times higher 2^nd^ Ab concentrations. The addition of 2^nd^ Abs without sHER2 did not result in a measurable signal.

In an actual assay recipe, a fixed concentration of added 2^nd^ Abs needs to be selected. Here, our chosen 2^nd^ Ab concentration of 25 nM allows maximal and roughly linear signal amplification within a measurement range up to about 5 nM sHER2 (the limit was obtained from the observed signal saturation at about five times the analyte molecule concentration).

Furthermore, the assay can be optimized by carefully choosing the best suited antibody pairs. To that end, we tested all combinations of Ab-a and Ab-b as primary and secondary antibodies. Corresponding measurement results are shown in the Supplementary Table S1 and indicate that the application of Ab-b as both primary and secondary antibody resulted in maximal measurement signal (case A). This is due to the polyclonal nature of Ab-b and the associated higher binding affinity compared to monoclonal antibodies. Consequently, when Ab-a is applied as primary antibody instead of Ab-b (case B), less antigen binds to the nanoprobes, resulting in lower signal on adding Ab-b as secondary antibody (case B vs. case A). As expected, as only a single epitope is targeted, the application of the monoclonal antibody type Ab-a as both primary and secondary antibody resulted in the lowest measurement signal (case D). When combining Ab-a and Ab-b, the signal was substantially larger for applying Ab-a as primary and Ab-b as secondary antibody (case B) as vice versa (case C). This we attribute to steric hindrance of the epitope targeted by the monoclonal Ab-a when the antigen is bound to the nanoprobes via primary antibody Ab-b. Based on these results, all subsequent measurements were carried out with Ab-b both as primary and secondary antibody (case A).

The next aspect to be addressed in order to optimize the assay for complex sample solutions like saliva and serum is the determination of the optimum dilution factor. Dilution is an important aspect of our measurement approach as the structure and the composition of the protein corona depends on the types and the concentrations of the proteins present in the sample solution and, therefore, on the sample dilution^17^. In addition, the nanoprobe rotation is also influenced by the concentration of non-bound proteins in solution, thereby affecting the drag torque and measured phase lag^18^. Consequently, by adjusting the dilution factor, the measurement signal can be maximized. To that end, different saliva dilutions were tested. Here, we have chosen a high initial concentration of sHER2 of 10 nM spiked into pure saliva to ensure that the concentration after dilution remains high enough to be easily detected. On the other hand, the initial concentration was chosen low enough to guarantee less than 5 nM even in low sample dilutions for optimal signal enhancement by the 2^nd^ Abs as shown above. The obtained measurement signals at different dilutions of saliva are shown in Supplementary Table S2. A dilution factor of 10 resulted in the highest measurement signal and was, thus, chosen in the following. For best possible comparability of serum and saliva samples, the same dilution factor was chosen also for serum samples.

Based on the information gained by the results presented above, we conducted phase lag difference measurements in dependence of the analyte concentration in solutions of pure buffer, serum, and saliva. For all these measurement series, we determined the relevant limits of detection.

**Figure 2:**
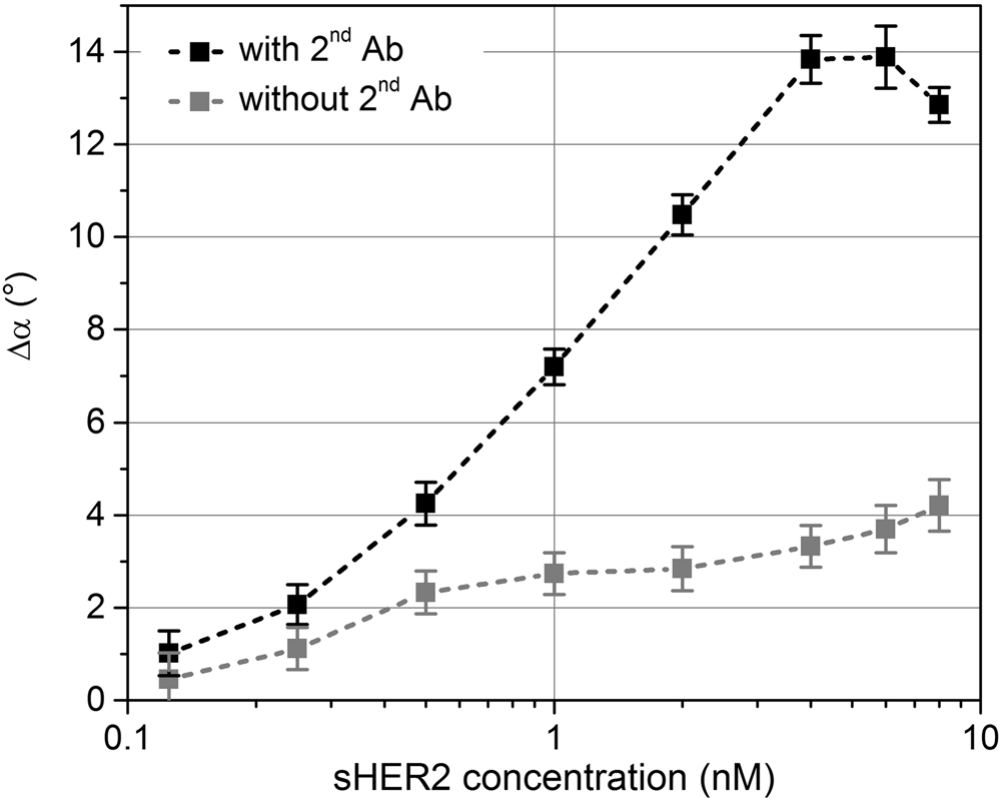
Phase lag difference signal Δα in pure buffer solution and its dependence on the antigen concentration. Measurement signal with (black curve) and without (grey curve) the addition of 2^nd^ Abs.

Figure 2 shows the measurement results for the sHER2 assay conducted in pure buffer solution. The phase lag difference signal Δα clearly increased with increasing analyte concentration up to a saturation level, and was substantially larger when 2^nd^ Abs were also added. In this case, the signal saturated at about 5 nM sHER2 concentration, and decreased for higher concentrations. This decrease is due to the now non-optimal ratio of 2^nd^ Abs to analyte molecules (see above). The achieved limits of detection were about 400 pM for the assay without 2^nd^ Ab addition and about 170 pM after addition of 2^nd^ Abs.

Finally, we conducted assay measurements in 10-fold diluted solutions of spiked serum and saliva with addition of 2^nd^ Abs. Generally, the results compared well with the ones obtained with 2^nd^ Ab addition in buffer solution. Specifically, the signals again rose up to a saturation level of about 5 nM sHER2 concentration. While in diluted serum, we deduced a limit of detection of about 470 pM, in diluted saliva samples sHER2 could be detected down to about 110 pM. The reason for the better measurement sensitivity in saliva compared to serum we attribute to different protein composition of serum and saliva samples and the difference in total protein content^15, 19^. Specifically, the lower reference phase lag in saliva allowed for a larger relative phase lag increase due to analyte and 2^nd^ Ab binding^12^. We ascribe the slightly better detection limit in diluted saliva samples compared to buffer solution to adsorption of tertiary proteins to the bound 2^nd^ Abs, which further increases the signal.

**Figure 3:**
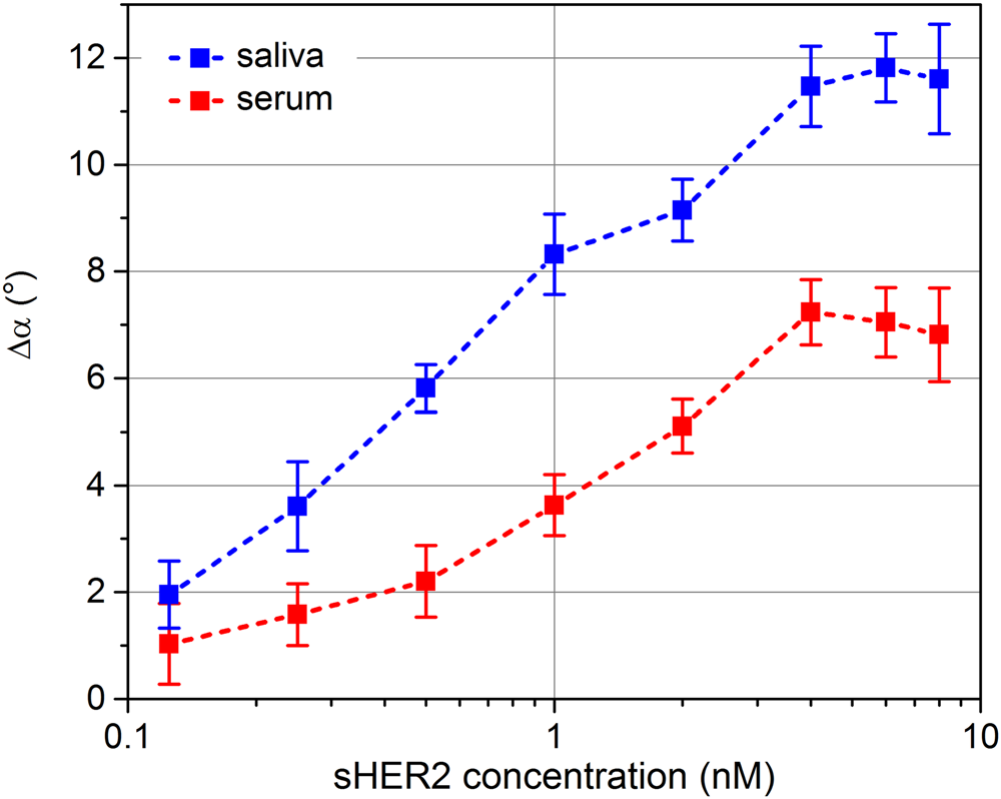
Phase lag difference signal Δα in 10-fold diluted solutions of spiked serum and saliva and its dependence on the antigen concentration. Measurement signal in saliva (blue curve) and in serum (red curve) under addition of 2^nd^ Abs.

## Conclusions

In this work, we demonstrated that our previously introduced nanoprobe-based homogeneous biosensing approach is also feasible to specifically detect analyte proteins in complex sample solutions (i.e. serum and saliva). While in buffer solution, direct analyte quantification is possible, the formation of a protein corona in complex samples screens the direct measurement signal. Consequent, the increase in hydrodynamic diameter due to antibody-mediated binding of target protein cannot be distinguished from non-specific adsorption (i.e. the formation of a protein corona). However, upon addition of secondary antibodies to the sample solution, there again is a specific further increase in hydrodynamic nanoprobe size, which results in a detection signal. Thus, what at first glance seems to be a disadvantage, can in fact be exploited to conduct *in*-*situ* reference measurements by simply not adding 2^nd^ Abs, which is an important feature for PoC testing environments.

We systematically elaborated measurement conditions to determine sHER2 analyte protein directly in spiked complex sample solutions by establishing a homogeneous sandwich assay directly at the surface of our dispersed nanoprobes. Recalculated to undiluted samples, our sHER2 limit of detection currently is about 4.7 nM in serum and 1.1 nM in saliva, which is about one order of magnitude above the clinical cut-off value for serum^14^, and, due to the substantially lower concentration, about two orders of magnitude above the clinical cut-off value for saliva^15^. However, our results demonstrate that by advancing the nanoprobe assay conditions, the detection limit can still be improved substantially. For example, in buffer solution we observed a significant enhancement of the signal and detection limit by introducing 2^nd^ Abs, which can be expected to further improve by applying labelled 2^nd^ Abs to bind large molecular weight biopolymers. In addition, while we tested some antibody combinations and observed a substantial effect on our measurement signal, there is a wide variety of different antibodies available to determine the best-suited pair.

In summary, for our simple mix-and-measure type homogeneous biosensor we succeeded to overcome a major hurdle that nanoparticle biosensors often suffer from, which concerns signal screening by protein corona formation. This opens up numerous applications in protein biomarker detection, specifically in PoC settings where resources are limited and ease-of-use is of essence.

## Methods

### Nanoprobe preparation

The nanoprobe preparation comprises the synthesis of bare Co nanorods (NRs), which were covered by a noble metal shell of Au and Pt according to already published procedures^20^. Afterwards, the NRs were transferred to aqueous solutions by over-coating them with an amphiphilic polymer as described earlier^10, 21^. The functionalization of the NRs with antibodies (Abs) was achieved by carboxy-amine linker chemistry. To that end, we employed EDC (N-(3-(dimethylamino)propyl)-N′-ethylcarbodiimide hydrochloride) and S-NHS (N-hydroxysulfosuccinimide sodium salt) at ratios of 1 × 10^4^ EDC molecules and 3 × 10^4^ S-NHS molecules per NR. The NR concentration was determined by inductively coupled plasma mass spectrometry and by the geometric NR dimensions determined by transmission electron microscopy as described elsewhere^10^. We employed a NR concentration of 730 pM and 2-(N-morpholino)-ethanesulfonic acid (MES) buffer solution at a concentration of 50 mM and at a pH of 5.5 throughout the whole functionalization procedure. In a first functionalization step, the NRs were incubated with EDC and S-NHS for 15 min at room temperature (RT) and then dialyzed against MES for 30 min at RT to reduce the concentration of EDC and S-NHS. The dialysis was performed with a Float-A-Lyzer G2 dialysis device with an approximate molecular weight cut-off value of 1000 kDa. Afterwards, the Abs diluted in MES were added at a volume comparable to the NR/EDC/S-NHS solution at a ratio of 200 Abs per NR, and the whole solution was incubated for 100 min at RT. Abs applied here were either trastuzumab in the form of the Herceptin therapeutic drug (Ab-a) or commercial HER2 antibodies (Ab-b) purchased from R&D Systems under catalogue number AF1129. Here, Ab-a is a monoclonal antibody, while Ab-b is of polyclonal nature. In a next step, 100 bovine serum albumin molecules per NR were added, followed by another incubation step overnight at 4 °C to block any remaining binding sites. Finally, to remove all unbound reagents, the functionalized NRs were dialyzed against MES at a pH of 5.5 for 48 h at 4 °C with a change of the dialysis solution after 20 h.

### Sample preparation and measurement conditions

The employed serum (pool of healthy male individuals) was obtained commercially from Sigma-Aldrich (product number H4522), while unstimulated whole saliva was collected in the morning from a single healthy male individual who refrained from eating before sample taking. The non-invasive saliva sample has been donated by a staff scientist of AIT following informed consent and approval by the Local Ethics Committee of the City of Vienna^22^. All methods were performed in accordance with the relevant guidelines and regulations. Saliva was filtered by a Whatman glass fibre syringe filter with a nominal pore size of 0.45 μm and stored in the fridge until usage. Filtering of the saliva was necessary to reduce the viscosity by removing glycoproteins and larger molecules without losing analyte molecules^23^. This procedure allows for preparing samples of a viscosity level comparable to serum by a very simple way suitable for PoC analysis.

The measurement buffer (MB) at a pH value of 7.4 was composed of 10 mM 4-(2-hydroxyethyl)piperazine-1-ethanesulfonic acid (HEPES) sodium salt, 150 mM NaCl and 0.05% v/v Tween 20. Samples spiked with sHER2 were prepared by mixing the MB or dilutions of serum/saliva (diluted by the MB) with the sHER2 antigen at a chosen concentration. Next, the nanoprobes were added to the sample solution. After an incubation time of 30 min at RT, the secondary antibodies diluted in MB (or volume-equivalent MB solutions for samples without added 2^nd^ Abs) were added, and the whole sample was incubated for another 60 min at RT. The overall sample volume amounted to 240 µl, and the nanoprobe concentration was 10 pM. Measurements were conducted at a RMF amplitude of 10 mT and a rotational frequency of 1 kHz. These parameters are chosen for optimal measurement signals to detect target proteins by our specific nanoprobes^10, 24^. Phase lag differences were recorded with respect to a suitable reference value. For the measurements executed in buffer solution only, we employed reference samples comprising all reagents except of the analyte sHER2 molecules. In complex sample solutions of serum and saliva, the reference phase lag value was determined with samples containing no secondary antibodies. Addition of the secondary antibodies only without analyte molecules did not result in a measurable signal. The errors of each measurement were determined by the standard deviations of the reference and the respective analyte-spiked samples and the error propagation law.

For determining the limit of detection, we defined a phase lag difference threshold. To that end, we added 3-times the obtained error of the lowest spiked sHER2 analyte concentration (i.e. 0.125 nM) to the reference phase lag. Next, the assay results of the measured phase lag differences were fitted by a 4-parameter logistic fit model, and the limit of detection was obtained by using the threshold value in the fitting curve equation.

## Electronic supplementary material

Supplementary Information
