# Supplementary material for: Direct protein quantification in complex sample solutions by surface-engineered nanorod probes
Source: Sci Rep. 2017 Jul 6;7:4752. doi: 10.1038/s41598-017-04970-5 (PMC5500566; doi:10.1038/s41598-017-04970-5)
Supplement: Supplementary file 1 — Supplementary Information [file 41598_2017_4970_MOESM1_ESM.pdf]

Supplementary Information

Direct protein quantification in complex sample solutions by surface-engineered nanorod probes

Stefan Schrittwieser[1*], Beatriz Pelaz[2,a], Wolfgang J. Parak[2,3], Sergio Lentijo-Mozo[4,b], Katerina Soulantica[4], Jan Dieckhoff[5,c], Frank Ludwig[5], and Joerg Schotter[1]

[1] Molecular Diagnostics, AIT Austrian Institute of Technology, Vienna, Austria

[2] Fachbereich Physik, Philipps-Universität Marburg, Marburg, Germany

[3] CIC Biomagune, San Sebastian, Spain

[4] Laboratoire de Physique et Chimie des Nano-objets (LPCNO), Université de Toulouse; INSA, UPS, CNRS, Toulouse, France

[5] Institute of Electrical Measurement and Fundamental Electrical Engineering, TU Braunschweig, Braunschweig, Germany

*Corresponding Author: Stefan.Schrittwieser@ait.ac.at

Present Address:

[a] Centro Singular de Investigación en Química Biológica y Materiales Moleculares (CiQUS) y Departamento de Física de Partículas, Universidade de Santiago de Compostela, Santiago de Compostela, Spain

[b] NABLA Lab, Biological and Environmental Sciences and Engineering (BESE) Division, King Abdullah University for Science and Technology (KAUST), Thuwal 23955-6900, Kingdom of Saudi Arabia

[c] Diagnostic and Interventional Radiology Department and Clinic, University Medical Center Hamburg-Eppendorf, Hamburg, Germany

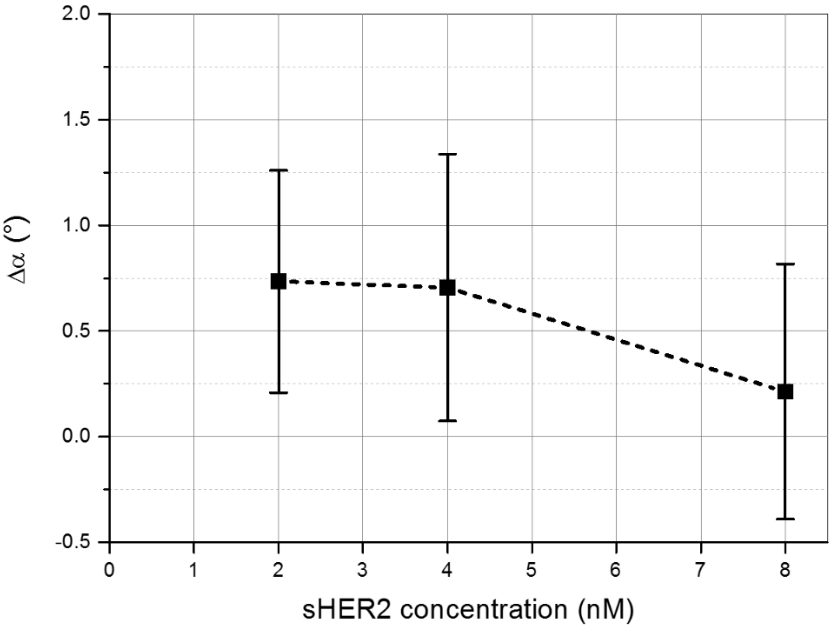

Figure S1. Measurement signal in 10-fold diluted serum samples at different spiked sHER2 analyte concentrations.

**Table S1.** Measurement signal for different antibody pairs in buffer solution.				
**case #**	**primary antibody**	**secondary antibody**	**phase diff. (°)**	**error phase diff. (°)**
A	Ab-b	Ab-b	10.48	0.44
B	Ab-a	Ab-b	8.63	0.39
C	Ab-b	Ab-a	3.64	1.01
D	Ab-a	Ab-a	0.56	0.61

**Table S2.** Measurement signal for different saliva dilutions.			
**saliva content (%)**	**sHER2 conc. (nM)**	**phase diff. (°)**	**error phase diff. (°)**
5	0.5	4.78	0.90
10	1	9.68	0.64
20	2	9.35	1.05
40	4	7.12	1.02
